# The effect of PCSK9 inhibitors on cardiovascular outcomes in diabetic patients: a narrative review

**DOI:** 10.3389/fphar.2026.1853705

**Published:** 2026-07-23

**Authors:** Abu-Baker Khalid Sharafeldin, Noor Elhuda Taher Aljundi, Abdullah Hussain, Ahmed J. Alaraibi, Alexandra E. Butler

**Affiliations:** 1 School of Medicine, Royal College of Surgeons in Ireland, Bahrain, Adliya, Bahrain; 2 School of Medicine, University of Jordan, Amman, Jordan; 3 Research Department, Royal College of Surgeons in Ireland, Bahrain, Adliya, Bahrain

**Keywords:** cardiovascular outcome, diabetes mellitus, evolucumab, inclisiran, lipid lowering therapy, PCSK9 inhibitor, statin

## Abstract

Cardiovascular events are a common occurrence in patients with diabetes mellitus. Multiple pharmacological interventions have been developed to reduce cardiovascular risk in this population; however, in this review, we focus on the role of proprotein convertase subtilisin/kexin type 9 (PCSK9) inhibitors in improving cardiovascular outcomes among diabetic patients. We provide an updated and comprehensive overview covering efficacy, safety, tolerability, and cost-effectiveness, while comparing these agents to the current standard of care, i.e., statins. Elevated low-density lipoprotein cholesterol (LDL-C) levels are strongly associated with adverse cardiovascular outcomes. By inhibiting LDL receptor degradation, PCSK9 inhibitors significantly enhance LDL-C clearance, leading to improved cardiovascular profiles. Evidence from large clinical trials such as FOURIER and ODYSSEY demonstrates consistent and promising reductions in major adverse cardiovascular events, even in diabetic subgroups. While the high cost and requirement for parenteral administration remain limitations, PCSK9 inhibitors represent a promising therapy for high-risk diabetic patients who fail to achieve lipid goals with conventional therapy and/or fail to adhere to the mainstay therapy due to adverse events.

## 1 Introduction

Cardiovascular diseases (CVDs) include a group of diseases that affect the heart and blood vessels (World Health Organization, 2025). These include, but are not limited to, coronary heart disease, cerebrovascular disease, peripheral arterial disease, congenital heart disease, deep vein thrombosis, and pulmonary embolism. Heart attacks and strokes happen as a result of obstructions that reduce the delivery of blood to the heart or the brain, but mostly as a result of atherosclerosis. Atherosclerosis is characterized by the accumulation of fatty deposits, resulting in a progressive reduction in arterial lumen perfusion; consequently, blood flow is blocked (World Health Organization, 2025). It is verified that the lipids and the lipoproteins play a part in CVD and represent a clear link with the leading cause of death worldwide. Atherosclerosis risk is enhanced in Type 1 and Type 2 diabetes mellitus (T1DM and T2DM) and hence is a key cause of death (Feingold, 2000a). With optimal glycemic control, lipid profiles in patients with T1DM are typically similar to those of non-diabetic controls. Contrasting with the characteristic lipid dysregulations found in even optimally controlled T2DM subjects, T1DM subjects have normal triglycerides, mildly increased low-density lipoprotein (LDL), and normal non–high density lipoprotein (HDL) and HDL cholesterol (HDL-c), with an increase in small, dense LDL particles. Both T1DM and T2DM patients with poor glycemic control experience worsening dyslipidemia, characterized by elevated triglycerides, reduced HDL-C, and a modest increase in LDL cholesterol (LDL-C) (Feingold, 2000a).

LDL is key to triggering atherosclerosis. When particles, composed of LDL, undergo oxidation and modification, they trigger an inflammatory cascade. Macrophages are enlisted that phagocytose oxidized LDL, generating foam cells (Jiang et al., 2022). Over time, the persistent buildup of lipids, combined with ongoing inflammation, promotes plaque formation, resulting in arterial narrowing and a loss of vascular elasticity. Rupture or plaque erosion leads to a thrombogenic substance, whereupon thrombi accrete to abruptly occlude blood perfusion, causing myocardial infarction (MI), ischemic stroke, and peripheral arterial disease (Loftus, 2011). An increase in LDL-C results from a decrease in hepatic clearance, accompanied by an increase in production, due to a reduction in hepatic receptors for LDL (Feingold, 2000b). The lipid profile is normal in T1DM patients with optimal glycemic control and is comparable to non-diabetic controls, with some reports noting modestly increased HDL-C levels (Feingold, 2000a). Epidemiological data indicate that dyslipidemia affects 30%–60% of patients with T2DM, making it a major contributor to their elevated risk of cardiovascular disease (Feingold, 2000a).

Proprotein convertase subtilisin/kexin type 9 (PCSK9) is a serine protease that interacts with the low-density lipoprotein receptor (LDLR) and directs the PCSK9–LDLR complex to lysosomal degradation, therefore reducing LDLR recycling and increasing circulating LDL-C (Pitts and Eckel, 2014). Genetic studies have clarified its clinical relevance by showing that gain-of-function mutations increase LDL-C and facilitate the development of coronary heart disease (CHD). Loss-of-function mutations reduce LDL-C and substantially lower cardiovascular risk (Ooi et al., 2017). PCSK9 inhibitors (PCSK9i) have therefore been engineered and demonstrated to cause significant reductions in LDL-C and LDL particle levels, resulting in substantial decreases in cardiovascular events (Qiao et al., 2022). Notably, among T2DM patients, the expression of PCSK9 is commonly upregulated, making it a significant factor contributing to the characteristic dyslipidemia, characterized by elevated triglycerides, reduced HDL-C, and small, dense LDL particles (Feingold, 2000a). This makes inhibition of PCSK9 a novel therapeutic strategy of particular relevance to reducing cardiovascular risk among diabetic patients (Feingold, 2000a). This review aims to provide a comprehensive and up-to-date analysis of the impact of PCSK9 inhibitors on cardiovascular outcomes among patients with diabetes, with additional emphasis on their tolerability, safety profile, and cost-effectiveness.

## 2 Methodology

A comprehensive search strategy using MeSH terms was carried out across databases including PubMed and Google Scholar. The search included research articles reporting clinical trial data published within the last 10 years to ensure the inclusion of the most current evidence. Articles included outside of the timeframe were only necessary to provide background information and explain underlying concepts or mechanisms. These were not used as the primary sources of clinical data explored in this review.

The search string used was (“Proprotein Convertase 9” [MeSH Terms] OR PCSK9 inhibitor OR evolocumab OR alirocumab OR inclisiran) AND (“Diabetes Mellitus” [MeSH Terms] OR diabetes OR diabetic patients) AND (“Cardiovascular Diseases” [MeSH Terms] OR cardiovascular outcome OR major adverse cardiovascular events OR MACE) AND (“Clinical Practice” [MeSH Terms] OR clinical use OR real-world use OR clinical efficacy). The search string was modified slightly to yield the required articles aligning with the specific themes and topics explored throughout the review.

Articles were selected based on their relevance to the review objectives and contribution to current evidence regarding the efficacy, safety, and clinical application of PCSK9 inhibitors in patients with diabetes mellitus. Conference abstracts, non-English publications, non-accessible full-text articles, letters to the editor, commentaries, and studies lacking sufficient clinical relevance or methodological rigor were generally excluded. Eligible publications included narrative reviews, systematic reviews, meta-analyses, randomized and non-randomized clinical trials, observational studies, and case reports, provided they contained information relevant to the objectives of this review.

The themes we aimed to explore in this review with regard to PCSK9 inhibitors include: the current clinical use of PCSK9 inhibitors, their efficacy in reducing cardiovascular risk, cost-effectiveness and cost–benefit considerations, recommendations from current clinical practice guidelines, dosing strategies, and adverse effects. Examining all these themes aids in providing a holistic approach in critically reviewing the use of PCSK9 inhibitors currently and its application for future use.

## 3 PCSK9 biology and mechanism of action

Cholesterol plays a crucial role in maintaining homeostasis and regulating normal physiological functions (Guo et al., 2020). If cholesterol levels are below or over the norm range, this correlates with multiple system diseases, including CVD, eye diseases, tumors, neurological and immunological diseases. Excess cholesterol, resulting from dysregulation of cholesterol metabolism, can induce oxidative stress, exacerbate inflammatory responses, impair autophagic function, and trigger apoptosis through multiple signaling pathways, collectively accelerating disease progression (Guo et al., 2020). Far from being merely a health concern, cholesterol is a fundamental lipid molecule indispensable to the daily functioning of living organisms (Cardoso and Perucha, 2021).

**FIGURE 1:**
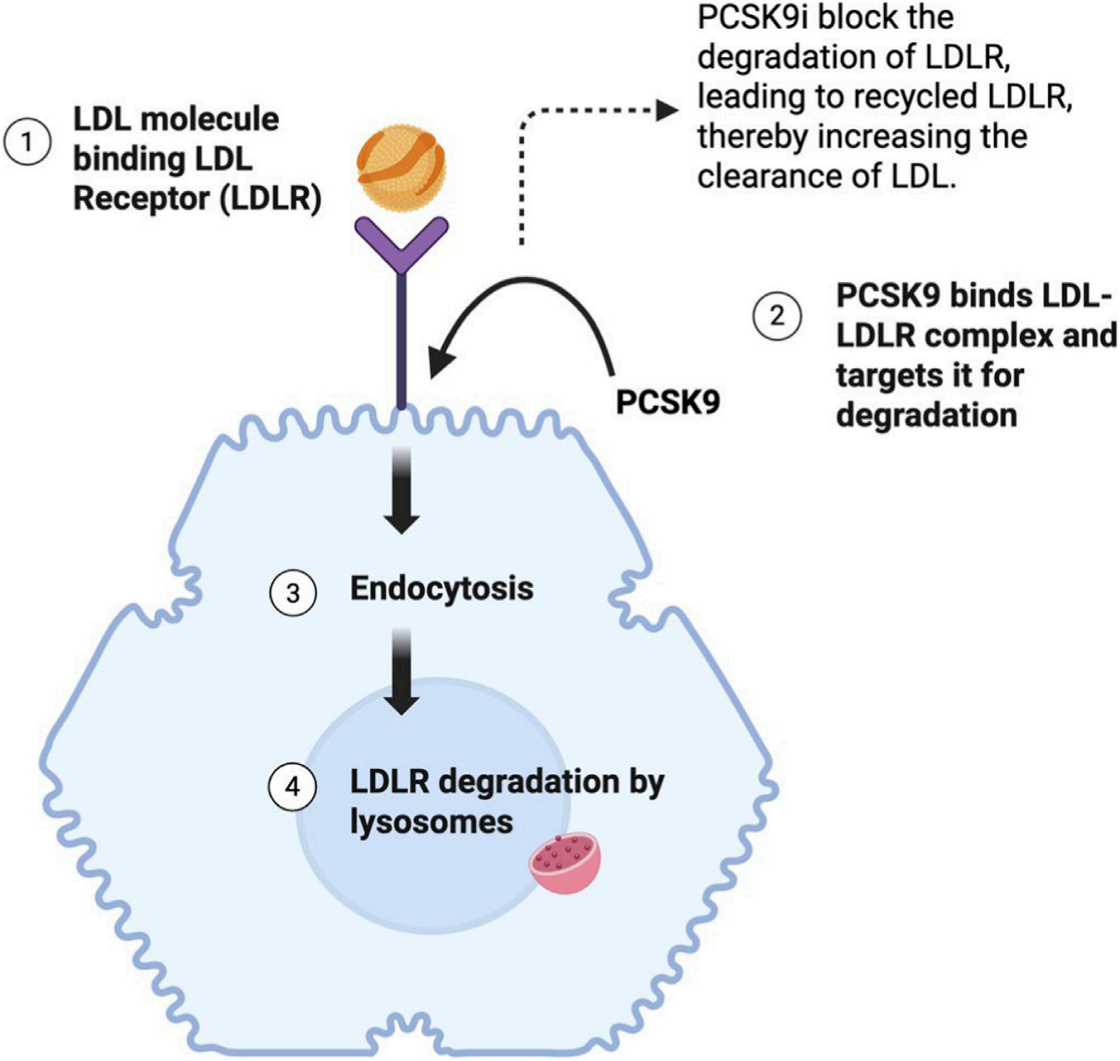
Mechanism of action of PCSK9 in normal physiology, and how PCSK9 inhibitors act to increase the clearance of LDL. Created with BioRender.com.

The PCSK9 protein is a key regulator of circulating LDL-C levels, as it negatively affects the recycling of LDL receptors (LDLR). On the hepatocyte plasma membrane, LDLR binds LDL particles, and the resulting LDLR–LDL complex is internalized through endocytosis (Loftus, 2011). Typically, a single LDL receptor (LDLR) can recycle back to the hepatocyte surface about 150 times. However, when secreted PCSK9 binds to LDLR on the cell surface, the internalized receptor is targeted for lysosomal degradation rather than recycling, thereby reducing the number of LDLRs available on the membrane. Inhibition of PCSK9 prevents this process, leading to increased LDLR expression on the hepatocyte surface and enhanced clearance of LDL-C from the bloodstream (Loftus, 2011). As a result, PCSK9 inhibition has emerged as a novel therapeutic strategy for lowering LDL-C, generally reducing cholesterol levels by 50%–60% (Hajar, 2019). Similarly, naturally occurring loss-of-function mutations in the PCSK9 gene are associated with lower LDL-C levels, reduced lifetime vascular exposure to LDL, and a significantly decreased risk of CHD (Loftus, 2011). Previous tests with other lipid-lowering therapies (LLTs) have also shown that diabetic mellitus patients receive at least an equivalent benefit from tight lipid control as do patients with other risk factors, or frequently benefit as much, if not more, than patients without diabetic mellitus, as demonstrated by the Improved Reduction of Outcomes: Vytorin Efficacy International Trial (IMPROVE-IT) analysis (Figure 1) (Loftus, 2011).

According to 2020 study, baseline PCSK9 levels were positively correlated with several cardiometabolic markers, including total cholesterol, LDL cholesterol, non-HDL cholesterol, and glycated hemoglobin A1c (HbA1c) (all p < 0.05) (Peng et al., 2020). During a median follow-up of 3.3 years, 103 cardiovascular events (8.4%) occurred. Patients who experienced events had significantly higher PCSK9 levels than those who did not (p < 0.05). Kaplan–Meier survival analysis revealed that patients with high PCSK9 levels had lower event-free survival compared with those with low PCSK9 levels (p < 0.05). Moreover, multivariable Cox regression analysis revealed that PCSK9 levels represented an independent predictor of major adverse cardiovascular events for diabetic patients (adjusted hazard ratio (HR): 1.361, 95% confidence interval (CI): 1.037–1.785, p < 0.05). Interestingly, when diabetic status was included as a stratum with high PCSK9 levels, there was a substantively increased risk of subsequent cardiovascular events developing (adjusted HR: 5.233, 95% CI: 2.546–10.757, p < 0.01) (Peng et al., 2020).

In diabetes mellitus, insulin resistance and hyperglycemia often result in increased PCSK9 expression that also decreases LDLR availability. Because atherosclerosis occurs slowly over decades, LLTs will frequently need to slow its development or prevent its occurrence. Recent LLT trials may not show clear cardiovascular mortality benefits because these effects often require longer follow-up; pooled statin trials showed a 12% reduction in all-cause mortality over 5 years, but little benefit during the first 1–2 years.

Assuming PCSK9i offers no mortality benefit within the first 1.5 years, and considering the short follow-up periods in the Further Outcomes Research with PCSK9 Inhibition in subjects with Elevated Risk (FOURIER) and ODYSSEY OUTCOMES trials, the expected reduction in cardiovascular death would be ∼6%, a figure consistent with findings from a recent meta-analysis of 39 randomized trials involving PCSK9i (Furtado and Giugliano, 2020).

Although statin therapy and genetic studies involving PCSK9 loss-of-function mutations linked to LDL-C reduction have shown a small but statistically significant increase in new-onset diabetes, current clinical data from alirocumab and evolocumab trials do not support a link between PCSK9i and impaired glycemic control. In ODYSSEY phase 3 trials (78–104 weeks), no changes were observed in fasting glucose or HbA1c among patients with diabetes, prediabetes, or normoglycemia at baseline (Handelsman and Lepor, 2018).

PCSK9 monoclonal antibodies (mAbs) are effective cholesterol-lowering medications that complement statins in achieving LDL targets when statins alone are insufficient (Kaddoura et al., 2020). They have been effective in cutting major cardiovascular events such as heart attack, stroke, unstable angina, and the requirement for coronary revascularization, but not mortality. They are generally safe, with injection-site reactions being the most frequent adverse effect; none have been established to cause memory or blood glucose problems (Kaddoura et al., 2020). The two PCSK9 mAbs that have received FDA approval are alirocumab (Praluent) and evolocumab (Repatha), administered by subcutaneous injection every 2–4 weeks, each of which has been associated with a 27% reduction in the risk of MI (Hajar, 2019). The fully humanized antibodies bind to circulating PCSK9, preventing it from mediating LDLR degradation (Sinning and Landmesser, 2020). This leads to an increased recycling of LDLR, such that LDL-C clearance is intensified as plasma LDL-C levels are significantly lowered. Inclisiran is a long-acting small-interfering RNA (siRNA) against PCSK9 mRNA, delivered subcutaneously with a N-acetylgalactosamine (GalNAc) conjugate which is a sugar molecule that promotes targeted uptake into hepatocytes. The drug achieves sustained LDL-C reduction by suppressing both intracellular and extracellular PCSK9. Its effects are prolonged such that the dosing schedule consists of only two injections per year. Clinical evidence confirms that Inclisiran substantially reduces LDL-C levels in individuals with diabetes, with no adverse effects on glycemic control, and possibly even a lower incidence of new-onset diabetes compared to statins (Sinning and Landmesser, 2020).

PCSK9i also represents an excellent choice for lifelong lipid management, particularly for patients with diabetes mellitus and a high cardiovascular risk. Clinical trials have demonstrated that PCSK9i significantly lowers LDL-C and reduces cardiovascular events in diabetic patients, without having an unfavorable impact on glycemic control or a higher incidence of new-onset diabetes (Jcpsp.pk, 2025). Thus, they are suitable for diabetic patients as well as those with dyslipidemia (Jcpsp.pk, 2025). Alirocumab is available as prefilled pens in either 75 mg or 150 mg strengths. The initial dose of 75 mg is recommended every 2 weeks. An every-four-week dosing of 300 mg is an appropriate alternative, with an escalation option every 2 weeks up to 150 mg if the reduction of LDL-C is insufficient. Evolocumab is a prefilled syringe or autoinjector of 140 mg/mL or a solution of 420 mg/3.5 mL in a single-use infusor (Pokhrel et al., 2025). Adults will receive 140 mg every 2 weeks or 420 mg per month, with pediatric use (≥10 years) licensed for familial hypercholesterolemia, dosing being titrated by disease severity. Inclisiran, a siRNA targeting PCSK9 mRNA via small-interfering RNA, is administered in a prefilled 284-mg/1.5-mL syringe, with baseline dosing and then again at 3 months, and then every 6 months thereafter. Such convenience, alongside effective LDL-C lowering and a reduction in cardiovascular events, illustrates the promise of PCSK9i as an effective treatment option for the secondary prevention of diabetic patients with established CVD (Jcpsp.pk, 2025).

## 4 Clinical trials and evidence

Many major clinical trials have been conducted to demonstrate PCSK9i efficacy in reducing LDL-C, and improving glycemic safety. This section aims to review these trials and the effectiveness of PCSK9i in diabetic populations (Table 1).

**TABLE 1:** PCSK9 inhibitor cardiovascular outcome trials.

Trial	Population (diabetic subgroup)	LDL-C reduction	CV outcomes	Mortality	Glycemic safety	Key findings
FOURIER	ASCVD, 27,564 pts; 36.6% diabetic (n ≈ 5,054)	Baseline LDL-C 92 mg/dL → 30 mg/dL; −59% at 48 weeks	Primary composite: HR 0.85 (95% CI 0.79–0.92, p < 0.001); key secondary (CV death, MI, stroke): HR 0.80 (95% CI 0.73–0.88)	No reduction; CV death HR 1.05 (95% CI 0.88–1.25)	No effect on HbA1c or FPG; no ↑ new-onset diabetes	Evolocumab reduced ischemic events in diabetics; greater absolute benefit due to higher baseline risk
ODYSSEY OUTCOMES	Recent ACS, 18,924 pts; 28.8% diabetes (n ≈ 5,444)	On-treatment LDL-C ∼31 mg/dL; −54.7% vs. placebo at 4 months	Primary composite: HR 0.85 (95% CI 0.78–0.93, p < 0.001); ARR in DM 2.3% (95% CI 0.4–4.2) vs. ∼1.2% in non-DM	All-cause mortality HR 0.85 (95% CI 0.73–0.98); DM subgroup underpowered	No ↑ new-onset diabetes; neutral HbA1c	Alirocumab reduced MACE; diabetics had greater absolute benefit
ORION-10 and ORION-11	ORION-10 (ASCVD, US, n = 1,561; 47.5% DM); ORION-11 (ASCVD or risk equivalent, EU/SA, n = 1,617; 36.5% DM)	Day 510: −52.3% (ORION-10); −49.9% (ORION-11); time-adjusted −53.8% and −49.2%	CV outcomes not yet available (ORION-4 ongoing)	NA	No effect on HbA1c/FPG; mild injection-site reactions (2.6%–4.7%)	Twice-yearly dosing provided durable LDL-C lowering in diabetics and non-diabetics

Abbreviations: MACE, Major Adverse Cardiovascular Events (definitions vary slightly by trial); ARR, absolute risk reduction; HR, hazard ratio; CI, confidence interval; ASCVD, atherosclerotic cardiovascular disease; ACS, acute coronary syndrome; DM, diabetes mellitus; LDL-C, Low-Density Lipoprotein Cholesterol; CV, cardiovascular; MI, myocardial infarction; HbA1c, Glycated Haemoglobin; FPG, fasting plasma glucose; pts, patients; wks, weeks; mo, months; US, united states; EU/SA, Europe/South Africa; NA, not available.

### 4.1 FOURIER (evolocumab)

The FOURIER trial randomized 27,564 patients with established atherosclerotic CVD (ASCVD) on statin therapy, approximately 36.6% of whom had diabetes mellitus (Sabatine et al., 2017a). Evolocumab achieved a placebo-corrected LDL-C reduction of 59% at 48 weeks, with similar efficacy in diabetic and non-diabetic participants. In the diabetes subgroup, evolocumab significantly reduced major adverse cardiovascular events (MACE), with HRs comparable to those without diabetes, and a greater absolute risk reduction because of higher baseline risk (Sabatine et al., 2017b). Cardiovascular and all-cause mortality were not reduced during the 2.2-year follow-up (Sabatine et al., 2017a). Importantly, evolocumab showed no worsening of HbA1c or fasting glucose and no increase in new-onset diabetes (Sabatine et al., 2017b). Long-term extension data from FOURIER-OLE confirmed persistent LDL-C reductions and no excess in new-onset diabetes (O’Donoghue et al., 2022).

ODYSSEY OUTCOMES enrolled 18,924 patients with recent acute coronary syndrome on intensive statin therapy. Prespecified glycemic-status subgroups included ∼29% with diabetes, 44% with prediabetes, and 28% normoglycemia (Schwartz et al., 2018). Alirocumab reduced LDL-C into the 30–50 mg/dL range across all strata, with consistent efficacy in diabetic patients. The relative reduction in MACE was similar across glycemic groups, but absolute benefit was nearly doubled in diabetics due to higher baseline risk (Schwartz et al., 2018). Glycemic safety was neutral, with no significant increase in new-onset diabetes or deterioration of glycemic parameters (Schwartz et al., 2018).

### 4.2 ODYSSEY DM-INSULIN and DM-DYSLIPIDEMIA

Two dedicated phase 3 trials evaluated alirocumab in patients with diabetes. DM-INSULIN included insulin-treated T1DM and T2DM patients with high cardiovascular risk, while DM-DYSLIPIDEMIA enrolled T2DM patients with mixed dyslipidemia. Alirocumab produced LDL-C reductions of 49%–51% in both studies, alongside improvements in non-HDL-C, apolipoprotein B (apoB), and Lipoprotein (a) (Lp(a)) (Leiter et al., 2019; Ray et al., 2019). Safety was favorable, with no evidence of worsening glycemic control or increased incidence of new-onset diabetes. Although not powered for cardiovascular outcomes, these trials directly confirm the efficacy and tolerability of PCSK9 inhibition in high-risk diabetic cohorts.

### 4.3 ORION program (inclisiran)

Inclisiran, a small interfering RNA targeting PCSK9 synthesis, was evaluated in ORION-9, ORION-10, and ORION-11. Across these trials, inclisiran achieved durable LDL-C reductions of ∼45–52% with twice-yearly dosing, with consistent effects in patients with and without diabetes (Ray et al., 2020). Safety was favorable, with mild injection-site reactions being the most common adverse events and no adverse glycemic effects reported. Cardiovascular outcomes in diabetic subgroups are not yet available; the ongoing ORION-4 trial will provide definitive evidence.

Across the major PCSK9 inhibitor trials, patients with diabetes experienced substantial LDL-C reductions (45%–60%) and consistent cardiovascular event reduction. FOURIER and ODYSSEY OUTCOMES showed relative risk reductions in diabetics comparable to non-diabetics, with greater absolute benefits owing to higher baseline risk. Mortality benefit in diabetic subgroups remains unproven, although alirocumab reduced all-cause mortality in the overall ODYSSEY OUTCOMES population. Dedicated trials (ODYSSEY DM-INSULIN and DM-DYSLIPIDEMIA) confirmed ∼50% LDL-C reductions and favorable safety in insulin-treated and dyslipidemic diabetic patients. Inclisiran demonstrated consistent 50% LDL-C lowering across glycemic strata, with outcome data pending.

Importantly, meta-analytic evidence in diabetics confirms an average of 48% LDL-C reduction, accompanied by significant improvements in non-HDL-C (−45%), apoB (−42%), Lp(a) (−28%), triglycerides (−12%), and modest increases in HDL-C (+6%) (Chen et al., 2023). PCSK9 inhibitors were glycemically neutral, with no impact on HbA1c or fasting glucose and no increased risk of new-onset diabetes (Chen et al., 2023). Collectively, this body of evidence positions PCSK9 inhibitors as potent, safe, and glycemically neutral lipid-lowering therapies that provide substantial incremental cardiovascular protection in high-risk diabetic patients.

### 4.4 Critical appraisal of the trial evidence

A critical appraisal of this evidence is nonetheless warranted. First, the pivotal outcome trials had relatively short follow-up a median of approximately 2.2 years in FOURIER and 2.8 years in ODYSSEY OUTCOMES which constrains the detection of a mortality benefit and likely explains the neutral effect on cardiovascular and all-cause mortality observed in FOURIER (Sabatine et al., 2017a; Schwartz et al., 2018). Second, the diabetes-specific findings rest largely on subgroup analyses that were not pre-powered for subgroup-specific outcomes; the dedicated DM-INSULIN and DM-DYSLIPIDEMIA trials established lipid efficacy and safety but were not powered for cardiovascular endpoints, and inclisiran cardiovascular outcome data in diabetic patients remain pending the ongoing ORION-4 trial (Schwartz et al., 2018; Leiter et al., 2019; Ray et al., 2019; Ray et al., 2020). Third, generalizability is limited because the trial populations were predominantly secondary-prevention patients with established ASCVD or recent ACS, so the evidence extends less readily to primary prevention (Jeswani et al., 2024). These constraints, together with reduced real-world persistence relative to clinical trials and the unresolved cost-effectiveness of PCSK9 inhibitors, should temper interpretation of the otherwise consistent efficacy data (Jones et al., 2016; Mercep et al., 2024; Basil, 2024).

## 5 Comparative efficacy and combination therapies

Given the novelty of PCSK9 inhibitors, it is essential to understand their advantages over similar, more commonly used drugs in diabetic patients.

### 5.1 PCSK9 inhibitors as an alternative to statins

Statins are the most common lipid-lowering drugs prescribed for diabetic patients and are well-documented for their efficacy in lowering cholesterol levels in both T1DM and T2DM patients. A meta-analysis comprised of 1466 T1DM patients and 17,220 T2DM patients revealed a 9% reduction in mortality for every 1 mmol/L decrease in LDL in diabetic patients. Diabetic patients displayed a reduction in MI (0.78, 0.69–0.87; p < 0.0001), coronary revascularization (0.75, 0.64–0.88; p < 0.0001), and stroke (0.79, 0.67–0.93; p = 0.0002) (Cholesterol Treatment Trialists, 2008). The study found that after 5 years, 42 fewer people per 1,000 on statin therapy with diabetes had major vascular events (95% CI 30–55) (Cholesterol Treatment Trialists, 2008). Other studies further displayed the efficacy of statins in diabetic patients. An observational study conducted over 5 years found that all statins were safe for hepatic and muscular function, and they all decreased LDL-C levels, with Rosuvastatin being the most effective (Bener et al., 2014).

Statins may also be effective in slowing the progression of diabetic nephropathy (DN). A recent meta-analysis found that statins significantly reduce albuminuria and urinary albumin excretion rates, especially in T2DM patients, but did not reduce glomerular filtration rate (GFR), serum creatinine, or blood urea nitrogen (BUN) (Shen et al., 2016). The major drawback of statins is tolerance, with side effects including muscle symptoms and/or elevated creatine kinase. Approximately 5%–10% of patients on statin therapy develop statin intolerance, leading to the use of other therapies such as PCSK9 inhibitors (Ahmad, 2014). However, it is important to note that there is currently no established evidence demonstrating the efficacy of PCSK9 inhibitors in improving diabetic nephropathy, and their use for this indication remains investigational. Statins are currently the predominant treatment option for dyslipidemia in diabetic patients, but they are associated with severe muscle-related side effects with a prevalence of around 7%–29%, and they can rarely cause an autoimmune myopathy even after the drug is discontinued (Stroes et al., 2015; Waters et al., 2016). Cognitive and memory problems are other reported adverse effects for patients on statin therapy (Gunta et al., 2023).

The effects of PCSK9 inhibitors in patients suffering from diabetes are promising. A recent meta-analysis comprising 8 RCTs following patients who were maximally treated with statin therapy found that major adverse cardiac events occurred in 8.7% of diabetic patients taking PCSK9 inhibitors compared to 11% of patients in the placebo group (Imbalzano et al., 2023). Similar meta-analyses have been conducted proving the efficacy of PCSK9 inhibitors as an adjuvant therapy or an alternative for statin-intolerant patients. For example, a meta-analysis studying the effects of PCSK9 inhibitors on diabetic patients already treated with statin therapy found a 48% reduction in LDL-C levels, noting more benefits in patients with lower HbA1c levels (Chen et al., 2023). The study also noted no safety concerns for the patients undergoing PCSK9 inhibitor therapy (Chen et al., 2023). Some theories have explored the potential for PCSK9 inhibitors in the treatment of DN, despite there not being any randomized controlled trials performed to investigate this. A recent review detailed the biochemical and inflammatory mechanisms behind PCSK9 inhibitors and the potential for their use as protective agents against DN (Bao et al., 2025). In short, PCSK9 inhibitors lower the risk of cardiac side effects in diabetic patients, and offer to be a useful alternative in those who are statin-intolerant, or those still unable to reach the LDL-C targets on the maximally tolerated therapy. The adverse effects of PCSK9 inhibitors are relatively mild. The most common adverse effects reported are an influenza-like illness, myalgia, back pain, and headaches (Gürgöze et al., 2019).

### 5.2 PCSK9 inhibitors + statins ± ezetimibe

PCSK9 inhibitors have been studied alongside ezetimibe and statins in patients requiring cholesterol-lowering therapy. PCSK9 inhibitors have shown good efficacy in reducing cholesterol levels in patients, as well as reducing the likelihood of adverse cardiac events. PCSK9 inhibitors, in conjunction with statins or ezetimibe, have also been studied with a meta-analysis investigating the effects of PCSK9 inhibitors + statin therapy and Ezetimibe + statin therapy reporting that adding PCSK9 inhibitors or Ezetimibe to statin therapy decreased the incidence of MI and stroke, but not the incidence of all-cause mortality or cardiovascular mortality (Khan et al., 2022). Specifically for diabetes, trials have shown that PCSK9 inhibitors, in conjunction with statins, may be more useful in lowering cholesterol levels than Ezetimibe in conjunction with statins in T2DM patients. The ODYSSEY DM‐DYSLIPIDEMIA randomized trial found that alirocumab was well tolerated and more effective in lowering non-HDL cholesterol than the usual care options, such as fenofibrate, ezetimibe, omega‐3 fatty acids, and nicotinic acid in patients who were maximally treated with statins (Ray et al., 2019).

PCSK9 inhibitors show greater efficacy when used in conjunction with statins than ezetimibe in conjunction with statins, and they show greater efficacy when given to statin-intolerant patients compared to ezetimibe, fibrates, and niacin.

### 5.3 Comparative efficacy against other PCSK9-targeting therapies

Alirocumab, evolocumab, and inclisiran all target PCSK9, but differ in mechanism of action, dosing, and cardiovascular effects. Alirocumab and evolocumab are monoclonal antibodies to circulating PCSK9 and similarly reduce LDL-C levels. Both agents have demonstrated reductions in major adverse cardiac events in major cardiovascular trials. In the FOURIER trial, evolocumab reduced LDL-C by 59%, and alirocumab reduced LDL-C by 55% in the ODYSSEY OUTCOMES trial (Sabatine et al., 2017b; Schwartz et al., 2018). Additionally, a recent meta-analysis demonstrated similar efficacy between these monoclonal antibodies in reducing major adverse cardiac events (Raone et al., 2026).

Inclisiran differs in mechanism of action by using small interfering RNA (siRNA) to suppress PCSK9 synthesis. The ORION program demonstrated an LDL-C reduction of approximately 50%, similar to the monoclonal antibodies, while being administered twice yearly (Ray et al., 2020). However, cardiovascular effects of Inclisiran have yet to be studied, and studies determining its long-term benefits are still being conducted (Ray et al., 2020).

## 6 Cost-effectiveness and safety, tolerability, and adverse effects

**FIGURE 2:**
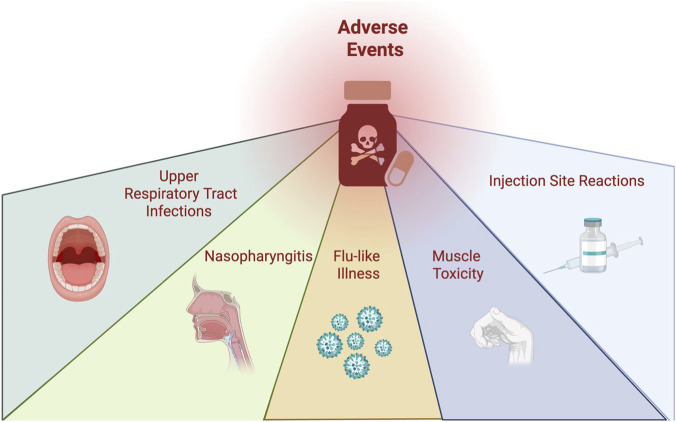
Adverse events of PCSK9i. Created with BioRender.com.

PCSK9 inhibitors, like other lipid-lowering therapies, are associated with adverse events. In a retrospective study, Zheng Feng et al. reported significant adverse events such as muscle toxicity, injection site reactions, and influenza-like illness (Feng et al., 2022). Trials evaluating PCSK9 efficacy also identified nasopharyngitis and upper respiratory tract infections as common side effects (Handelsman and Lepor, 2018). Injection site reactions were more frequently observed with alirocumab or evolocumab in all patient groups; additionally, patients with diabetes mellitus were found to have a lower incidence of this adverse event compared to those without diabetes (Figure 2) (Handelsman and Lepor, 2018). Furthermore, the long-term safety profiles of alirocumab and evolocumab were found to be comparable to control groups in patients both with and without diabetes (Handelsman and Lepor, 2018; Sabatine et al., 2017a; Jones et al., 2016).

In an article reviewing the cost-effectiveness of PCSK9 therapy, Iveta Mercep et al. concluded that, although the cost-effectiveness of PCSK9 inhibitors remains controversial, their benefit in high-risk patients is well established (Mercep et al., 2024). Early cost-effectiveness analyses suggested that the high price of PCSK9 inhibitors led to them being deemed non-cost-effective; and even with subsequent price reductions over the years, positive results have not yet been demonstrated (Mercep et al., 2024). On the contrary, a Swedish analysis reported in January 2022 that PCSK9 inhibitors can be cost-effective as part of combination therapy for secondary prevention of MI in very high-risk patients (Mercep et al., 2024; Landmesser et al., 2022).

## 7 Future directions and research gaps

PCSK9i are generally well-tolerated, but further research is needed to fully characterize their long-term safety profile, particularly in comparison with statins (Schwartz et al., 2018). Their most typical adverse effects are superficial injection site reactions. By contrast, safety issues related to neurocognitive effects, as well as those concerning glycemic safety, have not been established by extensive outcome studies. Although observational evidence suggests a possible increase in new-onset diabetes, randomized evidence remains negative. Still, the available evidence has limitations that could only be overcome by specifically designed longer-term studies of diabetic groups. In the ODYSSEY Outcomes trial, alirocumab was, for the most part, safe and well-tolerated, with an adverse effects profile comparable to that of the placebo (Schwartz et al., 2018). Similarly, ongoing trials such as SPECIAL with evolocumab will provide further clarity regarding the safety profile of PCSK9i in high-risk patients with acute coronary disease, as well as those with multivessel coronary disease (Jeswani et al., 2024). Together, these findings describe the favorable short-to mid-term safety profile of PCSK9i but also highlight the need for longer-term follow-up of diabetic patients to assess the contribution of PCSK9i to longer-term cardiovascular protection (Jeswani et al., 2024).

While statins remain the staple of primary prevention due to their low cost and robust evidence base, PCSK9i may be an effective adjunct for specifically selected patients, such as those who have familial hypercholesterolemia, T2DM, or high Lp(a), and who are unable to attain guideline-appropriate lipid levels or who are statin-intolerant (Khan et al., 2025).

One study explicitly addresses high-risk individuals within primary prevention populations, offering a narrow synthesis that differs from prior secondary prevention population studies. However, the majority of long-term cardiovascular outcome evidence continues to emanate from secondary prevention trials or multi-population analyses that demonstrate the need for future studies that formally contrast clinical endpoints in pure populations of primary prevention. Until such evidence is available, PCSK9i will deserve consideration as an individualized treatment that is selected in narrowly defined high-risk populations that are not responsive to usual treatment therapies (Khan et al., 2025).

PCSK9i and new gene-editing technologies open up new frontiers for lipid therapy by offering sustained reduction of LDL-C and the potential of lowering cardiovascular risk amongst diabetic patients (Basil, 2024). For example, gene editing treatment is an emerging and promising method that aims to provide a long-term therapeutic solution for hypercholesterolemia by targeting the gene for PCSK9, a key molecule in regulating cellular LDLR and, consequently, blood cholesterol levels. Gene therapy may provide a one-time, single-course, prolonged-course, or perhaps lifelong treatment for lowering LDL-C, an attractive solution, particularly for patients with familial hypercholesterolemia who require lifelong treatment commenced early in life (Basil, 2024).

Although effective and practical, the use of PCSK9i is limited due to adherence issues (Kosmas et al., 2018). For patients with diabetes, who generally tolerate several agents and injections, once-daily subcutaneous dosing can reduce long-term adherence. Clinic-to-real-life registry data approximate reduced persistence relative to clinical trials due to economic considerations, injection burden, and patient unfamiliarity (Kosmas et al., 2018). These constraints underscore the importance of education, convenient dosing options such as the twice-yearly regimen of Inclisiran, and greater accessibility as measures to preserve LDL-C lowering and cardiovascular protection in patients with diabetes (Kosmas et al., 2018).

## 8 Conclusion

In conclusion, patients with diabetes are at increased risk of cardiovascular disease, with elevated low-density lipoprotein cholesterol (LDL-C) being a major modifiable risk factor. PCSK9 inhibitors have emerged as an effective lipid-lowering therapy, reducing LDL-C levels by preventing the degradation of LDL receptors. Major clinical trials, including FOURIER, ODYSSEY OUTCOMES, and ORION, have demonstrated significant reductions in LDL-C and cardiovascular events with these agents.

PCSK9 inhibitors are generally well tolerated and provide an important treatment option for patients who are unable to achieve lipid targets with conventional therapy or who are intolerant to statins. They may also be used in combination with statins and ezetimibe to achieve greater LDL-C reductions and improved cardiovascular protection.

The 2026 multisociety dyslipidemia guideline supports the use of PCSK9 inhibitors in patients whose LDL-C remains above target despite maximally tolerated statin therapy and ezetimibe. With the adoption of more aggressive LDL-C goals, particularly in high-risk populations, the need for PCSK9 inhibitors is expected to increase. Although cost remains a consideration and generally places PCSK9 inhibitors after statins and ezetimibe in treatment algorithms, their substantial LDL-C–lowering efficacy and cardiovascular benefits support their use in appropriately selected high-risk patients. Overall, PCSK9 inhibitors represent a valuable therapeutic option for reducing cardiovascular risk in patients with diabetes (Blumenthal et al., 2026).
